# Sex Effects of Marijuana on Brain Structure and Function

**DOI:** 10.1007/s40429-016-0114-y

**Published:** 2016-07-07

**Authors:** Ariel Ketcherside, Jessica Baine, Francesca Filbey

**Affiliations:** Center for BrainHealth, School of Behavioral and Brain Sciences, University of Texas at Dallas, 2200 W. Mockingbird Lane, Dallas, TX 75235 USA

**Keywords:** Cannabis, Neuroimaging, MRI, Gender, Gendered treatment, THC, Sexual dimorphism

## Abstract

**Background:**

Tetrahydrocannabinol (Δ^9^-THC), the primary ingredient in marijuana, exerts its effects across several neurological and biological systems that interact with the endocrine system. Thus, differential effects of Δ^9^-THC are likely to exist based on sex and hormone levels.

**Methods:**

We reviewed the existing literature to determine sex-based effects of Δ^9^-THC on neural structure and functioning.

**Results:**

The literature demonstrates differences in male and female marijuana users on brain structure, reward processing, attention, motor coordination, and sensitivity to withdrawal. However, inconsistencies exist in the literature regarding how marijuana affects men and women differentially, and more work is needed to understand these mechanisms. While extant literature remains inconclusive, differentiation between male and female marijuana users is likely due to neurological sexual dimorphism and differential social factors at play during development and adulthood.

**Conclusions:**

Sex has important implications for marijuana use and the development of cannabis use disorders and should be considered in the development of prevention and treatment strategies.

## Introduction

Marijuana continues to be the most widely used illicit substance in the world [1, 2]. Similar to other substances of abuse [3], there are more male marijuana users in the USA (e.g., 54.1 %, [4]) than females historically; however, recent trends suggest that the number of female users is increasing, while the number of male users is remaining stable [5]. Interestingly, female users have been reported to develop cannabis use disorders (CUDs) more quickly after initiation of marijuana use than males (i.e., “telescoping”) [6–8], suggesting potential sex differences underlying the effects of Δ^9^-THC. These sex differences in the effects of Δ^9^-THC can be attributed to the interaction of the endocannabinoid and endocrine systems, as the endocannabinoid system is widely known for its modulatory role in endocrine functioning [9]. Alternatively, pre-morbid cognitive differences between males and females may also contribute to the sex differences observed following marijuana use. Nevertheless, these sex differences implicate the need for different prevention and treatment strategies for males and females (Table 1).

**Table 1:** Neurocognitive differences across male and female marijuana users

Authors	Modality	Participants	Putative difference between males and females
Anderson et al. (2010) [10]	Double-blind, placebo-controlled (active or placebo marijuana cigarette)	50 males, 35 females	No sex differences were observed.
Anderson et al. (2010) [11]	Attention, cognitive flexibility, time estimation, and visuospatial processing tasks	70 occasional marijuana users (50 % male)	No sex differences were observed, though the authors discuss a higher rate of study attrition in females.
Block et al. (1991) [12••]	Blood sample collection, hormone analyses, and substance use questionnaires	93 males and 56 females with either frequent, moderate, infrequent, or no marijuana use	No effect of chronic marijuana use on sex hormones (FSH, prolactin, LH, or testosterone) was found.
Block et al. (2000) [13]	Imaging: volume	18 adult marijuana users (50 % male) vs. 13 controls (46 % male)	Sex differences were detected in several brain volume measures.
Buckner et al. (2012) [14]	Multi-site, marijuana smoking history, MMM, MPS, SIAS	174 current marijuana users (57.5 % male)	Social anxiety in men was positively related to CUD problems and conforming and coping motives. Females demonstrated a positive correlation between social anxiety with social motives only.
Buckner et al. (2006) [15]	SCID DSM-IV-TR (SCID I/NP) and self-report questionnaires	123 undergraduates (40.7 % male)	Symptoms of social anxiety disorder were correlated with CUD symptoms in women only, and peer use of both alcohol and marijuana was found to moderate this relationship.
Cooper and Haney (2014) [16]	Data combined from four double-blind, within-subject studies using active vs. inactive marijuana	35 male vs. 35 female marijuana users	Women reported higher ratings of abuse-related effects relative to men under the active marijuana condition, but men and women did not differ in self-reported ratings of intoxication.
Copersino et al. (2010) [17]	Retrospective self-report measures	104 non-treatment seeking adult marijuana smokers (78 % male)	Females were more likely than males to report a withdrawal symptom (upset stomach, increased sex drive, marijuana craving).
Cousijn et al. (2012) [18]	Imaging: voxel-based morphometry (VBM)	33 heavy marijuana users (64 % male) vs. 42 controls (62 % male)	No interactions of either gray matter or white matter differences and sex was found between either of the groups.
Crane et al. (2013) [19••]	Neuropsychological tests	44 male vs. 25 female marijuana users	Earlier age of initiated use was related to less education, lower IQ, fewer years of maternal education, and poorer episodic memory in women only, but more lifetime marijuana use in men.
Felton et al. (2015) [20]	Self-report and BART task over period of grades 8–12	115 male and 89 female adolescents	An interaction of sex and disinhibition suggested that only males who self-reported greater disinhibition showed greater increases in their marijuana use.
Gillespie et al. (2011) [21]	Structured interviews on DSM-IV criteria of CUD	7316 adult male and female twins	Lower factor loadings for women suggest that legal problems may discriminate better among men.
Guxens et al. (2007) [22]	Cohort study, self-administered lifestyle questionnaire	1056 adolescents (52.2 % male)	Fewer factors predicting marijuana use were found in males than in females. Predictive variables reflecting type of school, family situation, and academic performance were present only among girls.
Hernandez-Avila et al. (2004) [7]	Self-report	271 substance-dependent patients (42 % male), 38 of those were marijuana-dependent (47 % male)	No sex effects on age of onset for marijuana users were found. Women who were marijuana-dependent reported less pretreatment years of regular marijuana consumption, compared to men. Women were less likely than men to be diagnosed with current marijuana dependence.
Johnson et al. (2015) [23]	Data from National Youth Risk Behavior Survey (YRBS) 1999–2013	115,379 adolescents (50 % male)	Sex differences were observed to substantially decrease over time for each race/ethnicity group.
Jones et al. (2008) [24]	Enzyme immunoassay assessment of blood	8794 adults (94 % male)	For DUID^a^ suspects, the number of men far exceeded that of women, and women were older than the men. Blood THC concentration was higher in men than in women.
Khan et al. (2013) [6]	Data from the National Epidemiologic Survey of Alcohol and Related Conditions 2001–2002	3297 US adults diagnosed with lifetime CUD (63 % male)	Women with CUD presented more mood and anxiety disorders and had an increased risk for externalizing disorders. Men with CUD had an increased risk of being diagnosed with an SUD, antisocial personality disorder, or a psychiatric disorder. Men with CUD were older at remission, used more joints, and reported more CUD symptoms than women.
Lisdahl and Price (2012) [25]	Neuropsychological tests	23 marijuana users (44 % male) vs. 35 controls (50 % male)	Female users were found to have an earlier age of onset for regular marijuana use. Male users also demonstrated a stronger relationship between both poor sequencing ability, psychomotor speed, and increased marijuana use, even though both male and female users had similar levels of past year marijuana use.
McDonald et al. (2003) [26]	Double-blind, placebo conditions, 7.5 or 15 mg THC capsule. Stop, Go/No-Go, delay discounting, and time estimation tasks	37 healthy recreational marijuana users (49 % male)	No significant sex differences in performance on the impulsivity measures were observed.
McQueeny et al. (2011) [27]	Imaging: sMRI	35 marijuana users vs. 47 controls (both groups 77 % male)	Female marijuana users had larger right amygdala volumes and more internalizing symptoms than female controls, while male users had similar volumes to male controls. For female controls and males, worse mood/anxiety was linked to smaller right amygdala volume, whereas more internalizing problems were associated with greater right amygdala volume in female marijuana users only.
Medina et al. (2009) [28]	Imaging: MRI	16 marijuana users (75 % male) vs. 16 controls	Female users presented more lifetime drinking episodes and symptoms of alcohol dependence. Male users showed smaller PFC volumes while female users displayed larger PFC volumes compared to their same-sex controls.
Noack et al. (2011) [29]	Internet survey of use characteristics	843 current cannabis-using students (70.6 % male)	Marijuana use with a water pipe was more often reported by males, while use before sleep was more often reported by females. When rating the social contexts of their marijuana use, women reported more use “with strangers” than men.
Pedersen et al. (2001) [30]	Longitudinal study of conduct and cannabis use	2436 adolescents (50 % male)	Conduct problems had an impact on marijuana initiation, with a noticeably stronger effect in females. For females, covert and aggressive conduct problems had robust effects, while in males, serious conduct had a moderate effect.
Pope et al. (1997) [31]	Visuospatial memory task	25 heavy marijuana users vs. 30 light marijuana users	Heavy marijuana-using women have impaired memory compared to light marijuana-using women, but there was no effect in men.
Price et al. (2015) [32]	Imaging: MRI	27 marijuana users (56 % male) vs. 32 controls (44 % male)	No significant sex interactions were found.
Roser et al. (2009) [33]	Double-blind, placebo-controlled cross-over study: psychomotor performance using finger-tapping test series	24 healthy volunteers (50 % male)	Males showed faster left-handed taps than females after ∆^9^-THC condition. Females showed greater variability in tapping speed after THC administration compared to placebo. No sex differences in the tapping frequencies under the placebo condition were found. Female subjects revealed a higher AIR-Scale score under ∆^9^-THC, but not under MJ extract. Overall, females performed worse than males for the left-hand tapping frequencies, demonstrated higher levels of plasma THC metabolites, and reported greater perception of intoxication compared to males.
Schepis et al. (2011) [34]	Cross-sectional statewide survey of adolescent risk behavior	4523 public high school students (48.2 % male)	African-American males and Caucasian females were more likely to use marijuana than their counterparts of the same race, while Asian females were less likely to use. Males with depression and anhedonia in the past year had greater odds of marijuana use in the past year as well. Overall, females also demonstrated more rapid transition from initiation to regular marijuana use.
Skosnik et al. (2006) [35]	EEG: visual function via SSVEP	17 marijuana users (59 % male) vs. 16 healthy drug naïve controls (38 % male)	No sex differences were observed in the marijuana group for any substance use data. A main effect of sex was observed, indicating that females displayed a larger SSVEP response. A sex-by-group interaction was observed at 18 Hz, indicating that marijuana use reduced 18 Hz spectral power in females, but not in males. Overall, these findings suggest that attention may be more impaired in male marijuana users.
Tu et al. (2008) [36]	Cross-sectional survey conducted in 2004	8225 students grade 7–12 (50 % male)	Aboriginal boys but not girls were more likely to use marijuana. Marijuana use was associated with higher school grade among boys, but not girls, and girls who used marijuana were more likely to report poorer mental health than boys.
Wetherill et al. (2015) [37••]	Imaging: fMRI	44 treatment-seeking, marijuana-dependent adults (61 % male)	Both sexes responded similarly to backward-masked marijuana cues > neutral cues, but for women, activity in the insula during this task correlated with MJ craving, while left OFC was inversely correlated with craving. For men, activity in the striatum during this task correlated with craving.
Zalesky et al. (2012) [38]	imaging: MRI axonal fiber connectivity	59 marijuana users (47 % male) vs. 33 matched controls (42 % male)	No significant sex differences or sex by group interactions were found.

^a^Driving under the influence of drugs

## Interaction Between Marijuana and Hormones: Mechanism for Sex Effects

The endocannabinoid system modulates the hypothalamic pituitary gonadal (HPG) axis. Animal studies have demonstrated that when Δ^9^-THC binds to cannabinoid receptors on gonadotropin-releasing hormone (GnRH) releasing cells in the hypothalamus, it has differential downstream effects in men and women. In females, there have been reports of Δ^9^-THC both stimulating and suppressing the secretion of luteinizing hormone, which not only indicates variable effects at different stages of the menstrual cycle [39–41].

Studies in humans have been inconsistent, however. For example, in chronic male marijuana users, decreased testosterone has been shown [42], but has not been replicated [39]. Further, one study found no effect of chronic marijuana use on sex hormones in males and females, including follicle stimulating hormone, prolactin, luteinizing hormone, or testosterone [12••]. In all, hormonal differences between men and women should be considered as a mechanism for differential effects of Δ^9^-THC on brain structure and function.

## Sex Differences on Brain Structures of Marijuana Users

Sexual dimorphism in the human brain is widely reported. For example, brain development is different between sexes such that total brain size peaks between 10 and 11 years of age in females, while total brain size peaks at 14 to 15 years of age in males [19••, 43]. Regarding overall brain tissue, an interaction between white and gray matter development and sex has been noted where prefrontal cortex (PFC) gray matter volume in females peaks 1–2 years earlier than in males, while males demonstrate greater age-related increases in white matter [44]. Brain regions specific to reward also develop differently between males and females. Amygdala volume increases in males as a function of androgen receptor density, while estrogen receptor density in the female hippocampus results in greater female hippocampal growth [45]. Thus, sex differences in brain structure may create a variable environment for Δ^9^-THC especially during periods of neural development when the brain is more vulnerable to structural and/or functional changes [4, 19••], such as in synaptic pruning. For example, in a study of adolescent (age 16–18) marijuana users, females had greater PFC volume than males, which was associated with impaired executive functioning, suggesting that sex moderates the relationship between marijuana use and PFC volume [28]. Similarly, another study found larger amygdala volumes in female adolescent marijuana users relative to non-users, which was not observed in male users [27]. Other studies, however, have not found similar interactions with sex [32].

In adults, while marijuana use has been associated with alterations in specific brain areas [46], differences between sexes have only been found on the whole brain level (e.g., total whole brain volume). In a study by Block and colleagues (2000), no effect of user vs. non-user status or sex on total brain tissue volume was found, although total intracranial volume, total intracranial tissue, cerebrospinal fluid (CSF), and combined cerebral gray matter were found to be higher in male marijuana users than in female marijuana users [13]. In a study using voxel-based morphometry (VBM), Cousijn et al. (2012) found no interactions of either gray matter or white matter differences and sex between groups [18].

In sum, the literature on sex-based differences in brain structure is limited. However, the existing studies suggest that male and female marijuana users have differences in brain structure, particularly in regions involved in reward processing. Differences in adolescent male and female users may be due to the sensitivity of this neural developmental period to Δ^9^-THC as alterations have been noted in mesocorticolimbic regions. However, there are inconsistencies in the literature. Future studies in adult marijuana users are needed to determine whether differences exist in localized areas of the brain.

## Sex Differences on Brain Function of Marijuana Users

The ubiquitous nature of the endocannabinoid system suggests that effects of Δ^9^-THC may span wide-ranging neural processes. In this article, we focus on each of the cognitive processes most widely examined in marijuana users and report sex-based differences demonstrated in the literature.

### Craving

Cannabinoids act directly on the nucleus accumbens triggering the release of dopamine in the mesolimbic “reward” circuit in the same manner as other drugs of abuse [47]. Though the mechanism is the same in males and females, evidence suggests differences in activation. For example, Cooper et al. (2014) demonstrated that despite no difference in levels of intoxication, females reported greater subjective positive effects than males (i.e., feeling “good,” and that they would “take it again”) [16]. Similarly, while both sexes experience subjective craving, Wetherill et al. (2015) demonstrated that this process might occur differently between treatment-seeking marijuana-dependent males and females [37••]. In their study, they used a backward masking of marijuana cues that allows for subconscious but not conscious processing of visual stimuli. Preliminary analyses showed greater response in the striatum, left hippocampus and amygdala, and left lateral orbitofrontal cortex (OFC) of females compared to males when exposed to the masked marijuana cues relative to masked neutral cues. Thus, females appear to exhibit greater cortical involvement in valuation of cues relative to males. Further analyses also revealed differences in correlations between neural response to marijuana cues and subjective craving with the most notable difference a negative correlation in the left lateral OFC in female users and an absence of a negative correlation in males. The negative correlation in females was interpreted as top-down cognitive control during exposure to cues, or the incorporation of previously learned patterns via higher-order (i.e., incorporating more information globally) brain regions in processing these cues. The lack of such processing in males suggests the lack of recruitment of these higher-order brain regions. In conclusion, the authors suggest that incorporation of top-down neural functioning may be a viable treatment approach, as pattern recognition may help those with CUDs (presumably males more than females) identify the deleterious effects of marijuana use [37••].

### Inhibitory Control

Impulsivity is a known risk fact for CUD [48] and may differ between the sexes in some components (i.e., sensation seeking) but not others (i.e., delay discounting) [49]. The concept of impulsivity is broad and encompasses several cognitive domains that characterize one’s ability to control one’s behavior. In a study of adolescent marijuana users, an interaction between self-reported behavioral disinhibition (a composite score of measures of impulsivity and sensation seeking) and sex was found, such that males with higher self-reported disinhibition were more likely to use marijuana than females (with higher self-reported disinhibition?) [20]. However, because impulsivity wanes through maturation, it would be important to determine if this trend continues in adulthood [50]. Currently, existing studies in adult marijuana users do not demonstrate sex differences in domains of impulsivity during acute Δ^9^-THC intoxication. For example, McDonald and colleagues did not find differences between adult male and female marijuana users on inhibitory control tasks including (i) the Stop Signal task, which measures the motor response inhibition to an ongoing motor response, (ii) the Go/No-Go task, which measures the ability to withhold a motor response to prepotent stimuli, or (iii) the Delay Discounting Task, which measures the cognitive ability to delay immediate, smaller rewards for larger, later rewards [26]. These inhibitory control functions may also be referred to as “stopping impulsivity” and “waiting impulsivity” respectively, and while both circuits implicate mesolimbic dopaminergic circuitry, the former involves motor regions while the latter involves top-down, cortical control of behavior [51]. The lack of sex effects in these domains suggests that Δ^9^-THC intoxication does not have unique sex effects on either motor or cognitive control [51].

In sum, the broad concept of impulsivity need to be disentangled in order to better understand the sex effects of marijuana on domains of impulsivity. However, in adolescents, general impulsive behavior is differentially linked to marijuana use between sexes.

### Motor Coordination

Given the abundance of cannabinoid receptors in brain motor control regions such as the basal ganglia and cerebellum, it is not surprising that studies have shown acute effects of THC on motor coordination and control, including how these may differ between the sexes. One task used to examine this is a finger-tapping frequency task that is designed to examine fine motor coordination. Using this task, Roser and colleagues (2009) found that males demonstrate significantly faster left-hand tapping than females after Δ^9^-THC administration, but not right (dominant) hand tapping [33]. Females in this study also reported higher subjective intoxication ratings and had higher concentrations of THC metabolites in their blood after being administered the same amount of Δ^9^-THC as males. The authors concluded that, while no effect was seen with the dominant hand, perhaps greater intoxication in females is related to greater “functional instability” in their non-dominant hand. Another study examining neuropsychological functioning in marijuana using adults found that male users demonstrated a stronger relationship between both poor sequencing ability, psychomotor speed, and increased marijuana use, even though both male and female users had similar levels of past year marijuana use [25].

Aspects of motor coordination can also be explored using driving tasks, although other cognitive processes such as attention likely confound overall performance. For example, Anderson et al. (2010) tested acute effects of Δ^9^-THC before and after smoking a marijuana or placebo cigarette [10] during a distracted driving simulator task. Participants who received the placebo cigarettes demonstrated learning effects wherein they exhibited improved performance the second time the test was administered, whereas participants administered marijuana cigarettes did not. However, no sex differences were noted in performance. The lack of sex differences in the driving task suggests that complex motor skills may not be as sensitive to sex effects of Δ^9^-THC as in those that measure fine motor coordination [10].

In sum, studies of motor coordination suggest that fine motor coordination differences between sexes may be related to degree of intoxication. Future studies should control for levels of intoxication and determine whether effects remain despite similar intoxication levels. More complex motor tasks should control for confounding effects of higher-order processes that may influence fine motor coordination.

### Memory

The literature widely supports the effects of marijuana on memory, particularly short-term memory in intoxicated individuals [52]. Studies focused on sex differences on memory functions in marijuana users suggest domain-specific effects. However, such findings have not been consistent. For instance, Pope et al. (1997) showed that heavy (smoked 29 out of the past 30 days) marijuana using females had impaired memory of visual checkerboard patterns compared to light (smoked one out of the past 30 days) marijuana using females, whereas no difference between heavy- and light-using males were found following a supervised abstinence period of at least 19 h. In addition, no difference was found between the sexes (i.e., heavy-using women vs. heavy-using men, or light-using women vs. light-using men) [31]. On the contrary, in a study examining acute effects of marijuana, Anderson et al. (2010) did not find a difference in visuospatial processing between sexes [11]. Similar to motor coordination, inconsistencies in differences in memory performance could be due to differences in levels of intoxication.

### Attention

While impaired attention has been documented in marijuana users [53], only one study to date documents differences between the sexes. Skosnik and collegues (2006) used electroencephalography (EEG) to examine attention via the steady-state visual evoked potential (SSVEP), particularly the N160 response, in current (at least one use per week) marijuana users. This response to visual stimuli is thought to measure attention by means of visual processing. Overall, the N160 response was lower in marijuana users compared to controls. Additionally, it was found to be lowest in the male marijuana users [35]. This suggests that attention may be more impaired in male marijuana users than in female users. However, two caveats were noted. First, males reported using 11.2 joints per week on average, which is greater than females, who averaged 9.3. Second, females’ menstrual cycle was not documented, which may affect visual processing [54].

There is limited literature suggesting general differences in attention between males and females, particularly on memory performance during divided attention [55], and during attention related to visual motor processing [56]. In light of this, given marijuana’s known general effects on attention, future work should examine potential differences between men and women both during acute intoxication and after chronic marijuana use.

## Conclusions and Future Directions

Although the number of studies that directly examine sex differences in marijuana users is limited (fewer than 30), the existing literature shows differences in brain structure and function. Differences in brain structure and function between male and female marijuana users show a complex picture that likely reflects the complicated sexual dimorphism that occurs in all biological systems. Thus, differential effects of marijuana based on sex may be different on multiple levels.

Our review revealed inconsistencies in the existing literature, which could be attributed to a number of factors. First, there may be underlying risk factors that are independent of sex. For example, risk factors for CUDs may be more prevalent within the subpopulation of women who do use marijuana and thus contribute toward CUDs. This is supported by the greater hedonic responses to marijuana reported by female users. However, the neural processes involved in this etiology are complex and require extensive further examination. Compounding the unique effects of marijuana on sex are multiple factors that have yet to be delineated. Studies have already begun to illustrate the importance of age of initiation of use [57]. Male and female brains develop at different rates and in different ways invariably resulting in differential effects of marijuana on each sex depending on age of exposure. Another limitation in studies of acute effects of THC in sexes is due to differences in subjective measures of intoxication. For example, D’Souza and colleagues identified variable reports in euphoria, perceptual alterations, feelings of anxiety, and disorganization of thoughts among acutely intoxicated participants. Variability between participants in these symptoms would likely confound effects of THC on outcome measures [58].

A final consideration worth noting is the self-selection in participants that are likely to confound study findings. As marijuana is still illegal in the majority of the USA, recruitment for marijuana using participants has its challenges. There may be differential effects of social norms on men and women that result in imbalanced representations between these sexes. One study revealed that “attempts were made to recruit equal numbers of men and women, however, fewer women expressed interest in participating in the study” [28]. Additionally, the women that do overcome aversion to admitting they use marijuana may demonstrate different personality traits than female marijuana users at large and could therefore be skewing the documented data. Thus, there may be limited generalizability in existing findings.

In conclusion, the differential effects of marijuana on the structure and function of male and female brains remains elusive. Some effects have been shown concerning attention, motor coordination, and impulsivity, but further work is needed to disentangle the mélange of variables affecting sex differences in marijuana users. Future directions should include controls for quantity of marijuana and ideally THC/CBD concentration, as well as females’ menstrual cycles, and age of initiation. Clinicians should consider the differences put forth by existing literature.
